# Economic Considerations on Costs and Pricing of Two Surgical Techniques for Treating Cranial Cruciate Disease in Dogs

**DOI:** 10.3390/ani13091505

**Published:** 2023-04-28

**Authors:** Annika Christina Wemmers, Szymon Pawlak, Nikola Medl, Jan Bokemeyer, Rolf Wagels, Oliver Harms, Holger Andreas Volk

**Affiliations:** 1 Department of Small Animal Medicine and Surgery, University of Veterinary Medicine Hannover, Bünteweg 9, 30559 Hannover, Germany; 2 AniCura Small Animal Referral Clinic, Alpenstr. 27, 87727 Babenhausen, Germany; 3 IVC Evidensia Small Animal Clinic Kalbach, Max-Holder-Straße 37, 60437 Frankfurt am Main, Germany; 4 Department of Information and Data Processing Service (IDS), University of Veterinary Medicine Hannover, Bünteweg 2, 30559 Hannover, Germany

**Keywords:** canine (dog), orthopedic disease, stifle surgery, cranial cruciate ligament rupture, Tibial Plateau Levelling Osteotomy (TPLO), Tibial Tuberosity Advancement (TTA), health care economics

## Abstract

**Simple Summary:**

Rupture of the cranial cruciate ligament in dogs is a very common condition in veterinary medicine. Surgical treatment is well established and several procedures provide very good clinical results. However, surgical treatment is associated with a high financial burden for patients’ owners. Veterinarians are bound to charging services according to a mandatory fee schedule, which gives a range in pricing. In this study, we examine the prices for two surgical interventions. We found that one technique is slightly more expensive; however, it also creates greater costs in the veterinary practice setting. Therefore, it is questionable whether the more expensive procedure provides a greater scope for profit. Pricing strategies are identified and may either be based on costs or a promise of quality in terms of a better clinical outcome.

**Abstract:**

In surgical treatment of cranial cruciate ligament disease in dogs, Tibial Plateau Levelling Osteotomy (TPLO) and Tibial Tuberosity Advancement (TTA) are commonly established procedures and have proven effective in restoring limb function. Unlike clinical outcome, economic aspects have not been studied as extensively. However, the surgical intervention poses an enormous financial burden on patients’ owners. In a veterinary practice setting, this study compares prices for TPLO and TTA and examines prices differences as well as potential cost drivers. Charges for veterinary treatments are based on the Gebührenordnung für Tierärztinnen und Tierärzte (GOT), which is mandatory for veterinarians in Germany but allows a certain range in billing. This study found that TPLO is charged at a higher price than TTA; however, this might not cover the additional costs of this procedure. The price is also associated with weight, heavier dogs being more expensive. The underlying strategies for pricing decisions may be based on costs, as efforts for TPLO and heavier dogs are higher in terms of a prolonged surgical time, the number of staff involved and in surgeons’ training. Price setting may also be based on a quality promise, suggesting better clinical outcome in a more expensive procedure. Future investigations should involve economic considerations and consider cost-effectiveness analysis when evaluating surgical treatment options.

## 1. Introduction

Cranial cruciate ligament (CCL) disease is one of the most common orthopaedic problems in canines [1] causing substantial restrictions in the joint’s functionality. Affected stifles suffer from clinical signs of lameness, progression of osteoarthritis, pain and instability [2,3,4,5,6]. Various surgical treatment options have been developed and established in treating this condition; however, there is no consensus on the most effective technique [7,8,9,10,11,12,13,14]. Whereas intra-articular and extra-articular surgical techniques aim to stabilise the stifle joint by miming the function of the intact cranial cruciate ligament and, therefore, reduce the cranial drawer [15,16,17,18], they have shown deficiencies in restoring full functionality [14]. The approach of altering the biomechanics in the knee joint by corrective osteotomies brought a new perspective into treating CCL disease [19,20]. The Tibial Plateau Levelling Osteotomy (TPLO) [8] and Tibial Tuberosity Advancement (TTA) [21] have both proven to be highly efficient treatment options [7,9,10]. Nevertheless, there remains a lack of profound kinetic and kinematic studies, as well as high-quality clinical trials, which makes a review and comparison of long-term clinical outcome challenging [7,9,10,12].

The high incidence of CCL disease [22] leads to an enormous economic impact, with an estimated cost of USD 1.3 billion for medical and surgical treatment in the US in 2003 [23]. To our knowledge, there is no comparable economic impact study in Germany. Veterinary surgeons in Germany are bound to charge their veterinary services according to the German fee regulation for veterinarians (“Gebührenordnung für Tierärzte und Tierärztinnen”, GOT) [24]. However, this legal requirement does not lead to a uniform price structure for all services, since the price items of the GOT can be interpreted variably and multiplied by a factor of one to three. This gives a certain freedom of design in pricing and, therefore, scope for different profit margins.

Profit generally is defined by revenue minus expenses [25]. Revenue is mainly generated by the veterinary work charged as fees for veterinary services according to the GOT as well as sale of medicine. Veterinary expenses consist of staff costs, costs for medication and materials, rent and leases, energy, insurance, depreciation, interest and other operating expenses [26]. These overall calculations must be considered in pricing individual veterinary services, such as orthopaedic surgeries.

There are several instruments to define an ideal pricing structure. Price can be determined by either cost-based or value-based approaches, depending on the pricing objectives. While, in a cost-based approach, the main leverage for the price is the costs in terms of expenses, the pricing in a value-based pricing suggests a certain quality [27].

The aim of this study was to determine the pricing structure of TTA and TPLO and, if possible, to identify drivers or influences on the total price. We hypothesise that there is a difference in price for TTA and TPLO. Furthermore, we will investigate price components and how they influence key economic figures. Finally, we will discuss factors on the decision-making process in pricing TTA and TPLO and how scientific evidence influences this process.

## 2. Materials and Methods

First of all, we must be careful using the term costs. Costs in terms of the GOT are the price charged to the owner. Therefore, it corresponds to the revenue of each surgical procedure. There are also costs that are direct and indirect expenses for the veterinarian related to the surgical treatment, such as staff, consumables, other materials and medication and general costs for running the veterinary practice. To differentiate the term cost, we will hereafter define the following terms:

- Price (P) = revenue of a single surgical procedure = charges billed to the patient owner;
- Cost (C) = expenses borne by the veterinarian related to the surgical procedure.

To determine the price of TPLO and TTA, we collected clinical and billing records from a veterinary hospital in Germany. The veterinary clinic regularly and alternatingly performs TPLO and TTA in dogs. The collected invoices from 2018 and 2019 were retrospectively reviewed alongside the clients’ records.

Inclusion criteria for billing records are:

- Clinical diagnosis of cranial cruciate ligament rupture as recommended by current standards [28];
- Surgical treatment of the CCL disease by either TPLO or TTA.

Exclusion criteria:

- Any comorbidities treated concurrently with CCL disease;
- Bilateral CCL disease, if surgical treatment is performed at the same time.

Patient data were blinded and included age at time of treatment, sex, castration status, breed and weight. Billing data include all charges billed on the date of surgical treatment. The price is calculated for both surgical treatments individually and compared between TPLO and TTA. All prices were net prices (19% value added tax not included). Patients were then divided into groups by weight (small dogs (group S): <15 kg; medium dogs (group M): 15–40 kg; large dogs (group L): >40 kg) and prices for all three groups were determined. In order to compare pricing for the different weight groups, we differentiated the population into small, medium and large dogs.

The most common breeds were mixed breeds (*n* = 30), Labrador Retrievers (*n* = 20), Bernese Mountain Dogs (*n* = 12), Boxers (*n* = 9), Beagles (*n* = 8), Old English Bulldogs (*n* = 6), Rottweilers (*n* = 5), Golden Retrievers (*n* = 5), Entlebucher Mountain Dogs (*n* = 4) and Australian Shepherds (*n* = 4). There were 28 males (17%), 48 neutered males (30%), 22 females (14%) and 64 neutered females (39%). Details on weight, age and sex at the time of surgery are given in Table 1. Weight and age were similarly distributed in TPLO group and TTA group.

We randomly selected 10 invoices for each of the two surgical techniques to analyse the individual price components. The invoices were based on the German fee regulation for veterinarians (GOT), which is mandatory for billing veterinary services. The services were differentiated into fees for veterinary services (“Gebühren für Leistungen”), compensation such as wayfare (“Entschädigungen”), expenses for postage or external services (“Barauslagen”) and the charges for medication and materials (“Entgelte für Arzneimittel und Verbrauchsmaterialien”) and multiplication factor (1–3). We set up an exemplary invoice for TPLO and TTA each to compare price components and we calculated the median price that is charged, respectively, for TPLO- and TTA-specific implants/materials. The invoices considered in our study were based on the version of the GOT that was valid in the period of investigation [29]. As a multiplication factor in our exemplary invoices, we use 1.44, as this is the mean factor that veterinarians applied (except for emergency services) in 2019 [30].

### Statistical Analysis

A Mann–Whitney U-Test was calculated to determine if there were differences in net prices between TPLO and TTA. Associations were considered to be statistically significant at *p* < 0.05.

## 3. Results

The search identified a total of 178 clinical records with diagnosis and treatment of CCL disease with TPLO (TPLO group) or TTA (TTA group). Surgeries were performed between 1 January 2018 and 31 December 2019.

Of the 178 records, we excluded 16 cases due to incomplete data (n = 10) or comorbidities that were treated at the same time as CCL disease (n = 6), which leaves 162 cases that met the inclusion criteria. A total of 75 stifles were treated with TPLO and 87 stifles with TTA. The net price for TPLO was EUR 1525.97 (median, range EUR 1169.77–1990.55). The mean net price for TTA was EUR 1475.60 (median, range EUR 1041.09–1731.47) (see Table 2). The net price between TPLO group and TTA group was significantly different (*p* < 0.05). The price for TTA is significantly lower than the price for TPLO.

Comparing the price for both TPLO and TTA between groups based on weight, we could see that price is higher when the dog’s weight is higher (see Table 3).

Price components are based on fees for veterinary services (“Gebühren für Leistungen”) and charges for materials and medication. Compensation such as wayfare (“Entschädigungen”) and expenses for postage or external services (“Barauslagen”) were not applicable in these invoices. The price components of the invoices are similar for TPLO and TTA and can be categorised into fees for veterinary services, materials and medication (see Table 4). The fees for veterinary services consist of a basic price multiplied with a factor of one to three and the quantity. Price components that vary between cases are the fee for infusion, monitoring of the patient during anaesthesia and anaesthesia time, as they depend on the duration of the procedure. The GOT provides a time factor that can be added to the basic fee. For materials, the median price for TPLO-specific materials/implants from the randomly selected 10 invoices is EUR 342.92 and for TTA EUR 416.24, respectively. Charges for medication can be divided into premedication, anaesthetics, analgesic and antibiotics and depend on the individual patients’ needs rather than the surgical technique.

## 4. Discussion

Our results show that the price does differ significantly between TPLO and TTA when comparing records from both procedures within one veterinary hospital. The price for TPLO is by around EUR 50 higher than for TTA, which might not be sufficient to offset the additional staffing costs required for TPLO, even if the material costs for TTA is more cost-intensive.

Possible reasons for the different pricing in the same surgical procedure may occur due to varying application of the GOT and cost structures. Therefore, it is important to take a closer look at how prices are designed in a veterinary practice setting.

**Figure 1:**
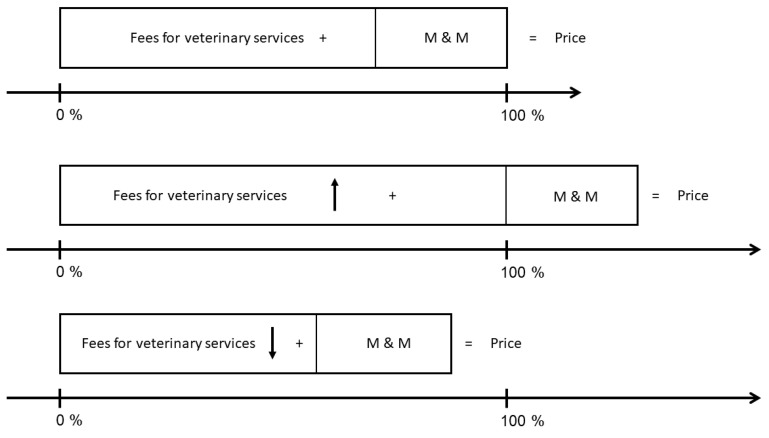
Price components and influence of alterations. The total price adds up from fees for veterinary services and medication and material (M & M). When fees for veterinary services rise because of the varying factor applied, the total price is higher with constant prices for medication and material. When fees for veterinary services drop, the total price is lower. The factor applied to the fees for veterinary services is the main driver in price setting when prices for medication and material are constant.

How price is set using the GOT. The price can be set by altering the factor between one and three multiplied by the basic fee for veterinary services. For example, the fee for surgery of the rupture of the cranial cruciate ligament without meniscal resection (No. B 3.12 a) can be charged between EUR 288.61 and EUR 865.83 [24]. Figure 1 shows the price components and the influence of the factor applied to the fees for veterinary services. The price consists of the fees for veterinary services (basic fee multiplied with the factor one to three) plus charges for medication and material. Charges for medication are very individual as medication is used as needed and tailored to the patient’s needs and weight. Material consists of procedure-specific material, such as implants, as well as consumables used before, during and after surgery.

The mean factor that veterinarians applied (except for emergency services) in 2019 was 1.44 [30]. We conclude that the main instrument for determining a price is the fees for veterinary services and the factor that is multiplied, plus a time factor if applicable. The price as the revenue needs to cover the costs as well as generate profit. Relative profit measured against net revenue is 29% in veterinary practices in Germany and varies with the size of the practice (measured by revenue), type of practice (individual or joint practice) and the kind of animals treated (small animals/companion animals or large animals/livestock) [26].

**Figure 2:**
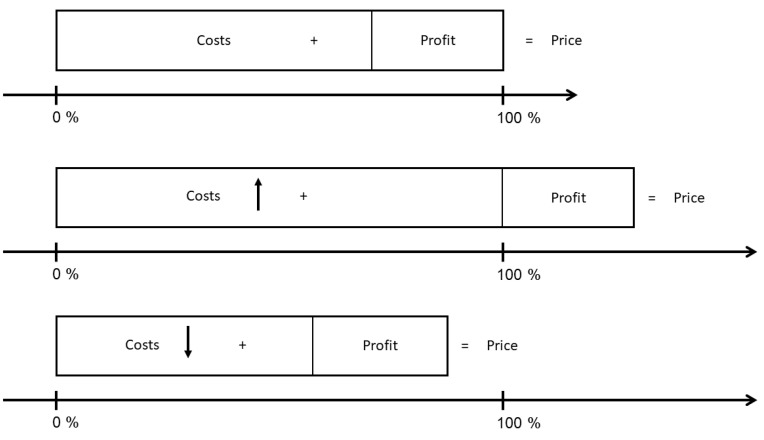
Influence of costs on the total price with constant profit. Price consists of costs and a certain profit margin. When costs for a procedure rise, the total price will be higher when retaining the same profit. When costs drop, the price will be lower.

How price is influenced by costs. Costs can be differentiated between general costs for running a veterinary practice and specific costs for a certain surgical procedure. The general costs for running a veterinary practice include staff costs, costs for medication and materials, rent and leases, energy, insurance, depreciation, interest and other operating expenses [26]. The costs have risen yearly, which made the need for a new version of the GOT become even more apparent [30]. The costs for a single product, in our example, a specific surgical procedure, differ in fixed and variable costs [25]. Fixed costs include proportionally general costs for running a veterinary practice but vary between procedures, for example, by certain tools and equipment or previous costs for the training of the staff. Variable costs vary depending on the number of staff needed to perform surgery, medication and materials used for the procedure as well as the duration of surgery. When costs for one of the two surgical procedures are higher than the other, price for this procedure will also be higher. Figure 2 shows the influence of the costs on the total price.

**Figure 3:**
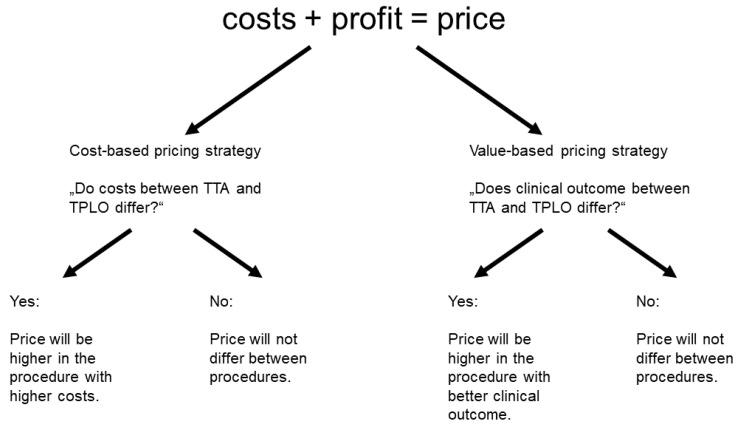
Pricing strategies based on cost or value. In a cost-based strategy, price is set by costs. When costs are higher in one procedure, pricing will also be higher. In a value-based strategy, price is set with a promise of quality. When clinical outcome is supposed to be better in one procedure, price will be higher.

How to decide which price to set for TPLO and TTA. The decision-making-process on pricing a surgical procedure can be based on a cost-based or value-based strategy. Therefore, we defined the following decision tree (Figure 3):

In a cost-based pricing strategy, price will be determined by the costs that come with a service or product [27]. Costs in a surgical procedure can vary on several factors (see above). Charges for TPLO-specific implants/material are relatively lower than for TTA-specific implants/material, which may leave a higher scope for profit. However, TPLO has a prolonged anaesthesia, as well as a longer surgery time compared to TTA [31,32,33], which binds capacities in terms of staff and, therefore, creates costs. At least one (for large dogs, rather two) veterinary nurse is needed for preoperative preparation and postoperative care of the dog. A veterinary nurse or veterinary assistant is present during surgery to monitor anaesthesia. The surgery is performed by an experienced veterinary surgeon and usually another veterinarian to assist. Literature estimates cost per minute of a treatment as EUR 2.25 [30]. With a median anaesthesia duration of 280 min for TPLO and 198 min for TTA [31], that generates costs of EUR 630.00 for TPLO and EUR 445.50 for TTA. We believe that the cost per minute of surgical treatments such as TPLO and TTA is higher than the weighted average of EUR 2.25, as surgery requires an above-average number of personnel and equipment. The calculation example gives an indication of the impact of treatment time on the costs of a procedure. When costs are higher for one of the two techniques, the price for the technique with higher costs will be set higher in a cost-based price strategy or be set at the same level in case costs do not differ.

Profitability. We found that the price for TPLO is around EUR 50 higher than for TTA, direct costs for TPLO-specific materials/implants are lower than for TTA and the cost structure for other materials and medication, as well as the number of staff needed, is similar, which may evoke the assumption that there is a higher scope for profit for TPLO. However, there is a striking impact of the surgical time on costs, as the duration for TPLO is much longer than for TTA given that the performing surgeons have a similar level of experience. The costs for staff needed for the procedure and the share of general costs for running a veterinary practice increase with extended surgical time. This increased effort must be reflected in price setting by applying a higher multiplication factor and/or adding a time factor, when possible. Furthermore, TPLO requires specific training and is supposed to be more difficult to learn, which leads to higher indirect costs for the surgeon’s education. Altogether, we believe that the price difference between TPLO and TTA must be significantly higher than EUR 50 in order to ensure TPLO’s profitability. In brief, TPLO is associated with higher costs due to the surgery’s duration and binding staff during this time, as well as the surgeon’s training.

We previously investigated on the clinical outcome of TTA and TPLO but were not able to clearly prefer one over the other. Both techniques provide a good functional outcome in terms of subjective and objective gait analysis, thigh circumference measurements, goniometry, joint stability and postoperative pain. TTA appears to have slower progression of osteoarthritis within six months after surgery, while TPLO results in lower complication rates, especially surgical site infections [10]. In a value-based pricing strategy, pricing correlates with quality and, therefore, a more expensive alternative suggests better quality [27]. If quality in terms of clinical outcome is more favourable in one of the surgical techniques, a value-based pricing strategy will price the more favourable option higher. The quality of a surgical procedure can also be defined by its versatility and limitations. TPLO allows a simultaneous correction of stifle deformities, such as varus, valgus and rotational deformities [34], and is recommend as the treatment of choice for large dogs (>15 kg), very active dogs, partial CCL ruptures and excessive tibial plateau slopes [2,35,36,37].

In order to evaluate the relationship between the outcome of the treatment option and its cost, there are various empirical methods of health economics. In human medicine, cost-effectiveness analysis is a successfully used technique to examine the cost of orthopaedic treatment in relation to the quality of treatment outcomes [38,39]. The question answered by these instruments is “Which option provides the greatest benefit at an acceptable cost?” Benefit can be expressed monetarily or in natural or physical units, or it can be converted to a quality-adjusted life year (QALY), which combines quantitative criteria (extended life) with quality of life generated [40,41]. This approach may be a helpful method to investigate pricing, especially in a value-based strategy.

There are several limitations to our study. We only compared data from one veterinary hospital because we wanted to rule out pricing differences that are accounted to a different pricing policy between two veterinary hospitals. However, we believe that the differences in prices become even more apparent when comparing prices between veterinary hospitals. Further research is needed to compare pricing structures between a large number of veterinary hospitals in order to confirm our results. With the rising importance of pet insurances in Germany, billing data from insurance companies may provide a valuable basis for this comparison. Moreover, prices do not represent the overall treatment cost in treating CCL disease. Additional treatment due to postoperative complications or the need for physical therapy and rehabilitation pose another financial burden on dogs’ owners. Further research may also focus on the overall treatment cost, which takes into account all costs related to the disease. Another limitation is posed on the retrospective character of our study. We did not discount invoices, as price rises of the GOT are not carried out regularly (yearly) and our study period only covers two years. Nevertheless, veterinary hospitals are able to enforce price rises any time by customising the factor applied to the basic fee. A prospective trial with tracking of possible price rises and corresponding discounts in the evaluation is needed to rule out this limitation.

## 5. Conclusions

Prices of surgical treatment of CCL disease by TPLO and TTA differ and further research on the underlying reasons for pricing decisions is recommended. Both procedures are an effective treatment option for CCL rupture and deliver excellent clinical results with minor differences in outcome parameters. TPLO is charged at a slightly higher price, which may indicate a higher scope for profit. However, there are differences in expenses for performing both procedures. TPLO is associated with higher expenses in the surgeon’s training, in TPLO-specific instruments, as well as a prolonged anaesthesia and surgery time, which creates costs in terms of binding staff and general costs for running a veterinary practice. Pricing decisions in veterinary medicine should be based on comprehensible profitability calculations and consider direct, as well indirect, costs for a certain procedure. More investigations are needed in order to evaluate cost-effectiveness and economic impact, as these types of studies are sparse in veterinary medicine.

## Figures and Tables

**Table 1:** Number of treatments, weight and age of operated dogs for TPLO and TTA.

Group	TPLO	TTA
No. of treatments	75	87
Weight (in kg)		
Median	29.8	30.0
Min.	6.0	7.0
Max.	53.0	75.0
Age (in years)		
Median	7	7
Min.	1	1
Max.	12	12

**Table 2:** Price for TPLO and TTA each. Price is significantly higher for TPLO compared to TTA (*p* < 0.05, Mann–Whitney *U*-Test).

Price (in EUR)			*p* Value
Median	1525.97	1475.60	0.0006
Min.	1169.77	1041.09	
Max.	1990.55	1731.47	

**Table 3:** Median prices for TPLO and TTA when comparing on weight. Group S < 15 kg, Group M = 15–40 kg, Group L > 40 kg. In both TPLO and TTA group, price is higher with increasing weight.

Group	TPLO	TTA
S	1467.80	1338.15
M	1560.30	1460.90
L	1762.60	1500.58

**Table 4:** Price components for TPLO and TTA; price setting according to the GOT. Fees for veterinary services are listed and the overall price (OP, in EUR) per fee is calculated by multiplying the basic price (BP, in EUR) with the multiplication factor (F, 1.44) and the quantity (Q). If applicable, a time factor (T*) is added. Prices for materials are divided into procedure-specific materials/implants and consumables. TPLO-specific implants/materials have a lower median price than TTA-specific implants/materials. Prices for medication are individual as they depend on the type of medication and the patient’s weight. Prices highlighted in grey are prices that depend on the procedure’s individual effort and costs and may, therefore, differ between TPLO and TTA.

TPLO					TTA				
Fees For Veterinary Services (Calculation: Basic Price (BP) × Multiplication Factor (1–3) (F) × Quantity (Q) = Overall Price (OP))									
Description	BP (EUR)	F	Q	OP (EUR)	Description	BP (EUR)	F	Q	OP (EUR)
X-ray, 1. and 2. image	32.07	1.44	2	92.36	X-ray, 1. and 2. image	32.07	1.44	2	92.36
X-ray, further images	19.24	1.44	2	55.41	X-ray, further images	19.24	1.44	2	55.41
Injection (subcutaneous, intracutaneous, intramuscular)	5.77	1.44	2	16.62	Injection (subcutaneous, intracutaneous, intramuscular)	5.77	1.44	2	16.62
Inserting venous catheter	15.39	1.44	1	22.16	Inserting venous catheter	15.39	1.44	1	22.16
Injection (intravenous)	7.71	1.44	2	22.20	Injection (intravenous)	7.71	1.44	2	22.20
Infusion, dog	12.84	1.44	T*	18.49 + T*	Infusion, dog	12.84	1.44	T*	18.49 + T*
Monitoring of anaesthesia or vital functions	38.48	1.44	T*	55.41 + T*	Monitoring of anaesthesia or vital functions	38.48	1.44	T*	55.41 + T*
Inhalation anaesthesia, intubation anaesthesia	38.48	1.44	T*	55.41 + T*	Inhalation anaesthesia, intubation anaesthesia	38.48	1.44	T*	55.41 + T*
Joint orthopaedic surgery: arthroscopy	128.27	1.44	1	184.71	Joint orthopaedic surgery: arthroscopy	128.27	1.44	1	184.71
Joint orthopaedic surgery: rupture of the cranial, caudal or both cruciate ligaments without meniscal resection with meniscal resection	288.61 or 352.76	1.44 1.44	1 1	415.60 or 507.97	Joint orthopaedic surgery: rupture of the cranial, caudal or both cruciate ligaments without meniscal resection with meniscal resection	288.61 or 352.76	1.44 1.44	1 1	415.60 or 507.97
Applying bandage (Robert Jones bandage)	19.24	1.44	1	27.71	Applying bandage (Robert Jones bandage)	19.24	1.44	1	27.71
**Materials**									
TPLO-specific materials/implants (Median prices from 10 billing examples)			EUR 342.92		TTA-specific materials/implants (Median prices from 10 billing examples)			EUR 416.24	
Consumables			Standard charge per orthopaedic surgery/individual use if applicable		Consumables			Standard charge per orthopaedic surgery/individual use if applicable	
**Medication**									
Premedication			Individual/as needed/per weight		Premedication			Individual/as needed/per weight	
Anaesthesia medication					Anaesthesia medication				
Analgesic					Analgesic				
Antibiotics					Antibiotics				

## Data Availability

Data are contained within the article.

## Abbreviations

BP	Basic price
C	Cost
CCL	Cranial cruciate ligament
F	Factor
GOT	Gebührenordnung für Tierärztinnen und Tierärzte
Kg(s)	Kilogramm(s)
L	Large
M	Medium
M & M	Medication and material
Max.	Maximum
Min.	Minimum
OP	Overall price
P	Price
Q	Quantity
QALY	Quality-adjusted life year
S	Small
T	Time factor
TPLO	Tibial Plateau Leveling Osteotomy
TTA	Tibial Tuberosity Advancement
